# Body Image Perception and Attempts to Change Weight among Female Medical Students at Mangalore

**DOI:** 10.4103/0970-0218.66886

**Published:** 2010-04

**Authors:** D Priya, K S Prasanna, S Sucharitha, Nafisa C Vaz

**Affiliations:** Department of Community Medicine, Father Muller Medical College, Mangalore, India

**Keywords:** Attempts to change weight, body image perception, body image satisfaction, body mass index, skipping meals

## Abstract

**Background:**

Assessing body image self-perception has used BMI as an indicator of nutritional status. The visual analogue scale is a highly effective instrument for assessing people’s level of dissatisfaction with their body weight while evaluating the perceptual component of body image.

**Objective:**

By knowing body mass index of female medical students, to find out their pattern of body image perception and any attempts done to change their weight.

**Materials and Methods:**

All the students residing in MBBS ladies hostel were included in this study and a questionnaire regarding body image perception, diet, physical activity and attempts to change weight was instituted. Their responses were collected, tabulated, analyzed and interpreted.

**Results:**

Among 147 study subjects, according to BMI, 25(17%) were undernourished while 111(75.5%) and 11(7.5%) were normally nourished and overweight respectively. 35(23.8%) of the subjects felt they were lean, 95(64.6%) felt they were normal and 17(11.6%) felt they were overweight. Regarding image satisfaction, 98(66.7%) of them were satisfied with their image and out of 49 who were not satisfied 30 (20.4 %) wanted to reduce weight. Skipping meals was practiced by 42 (28.6%) of subjects.

**Conclusion:**

About 75.5% of the study group were having normal BMI. Most of them perceived their image correctly regarding to their weight. Most of the underweight and all overweight females were not satisfied. Underweight females preferred to gain weight and overweight females preferred to lose weight.

## Introduction

Globally there are more than 1 billion overweight adults, at least 300 million of them are obese. Obesity and overweight pose a major risk for chronic diseases, including type 2 diabetes, cardiovascular disease, hypertension and stroke and certain forms of cancer. The key causes are increased consumption of energy- dense foods high in saturated fats and sugars and reduced physical activity.(1)

Obesity is perhaps the most prevalent form of malnutrition in developed countries. There is an increased awareness of the problem in recent years. It has been estimated to affect 20-40% of the adults and 10-20% of the children and adolescents in developed countries. Physical inactivity may cause obesity, which in turn restricts activity. This is a vicious circle. It is the reduced energy output that is probably more important in the etiology of obesity.(2)

The relationship between eating disorders and body image self-perception is well documented in the literature.(3) However, Leonhard and Barry(4) notes that studies on body image distortion and dissatisfaction have mostly focused on subjects with specific eating disorders (bulimia, anorexia, nervosa and morbid obesity). It has recently been described a similar body image distortion in normal weight individuals without any specific eating disorders.

Body image is an important element of the intricate mechanism of one’s own identity. Gardner(5) defines it as “the mental picture we have of our body’s measures, contours and shape; and our feelings related to these characteristics and to our body parts”. The subjective component of body image refers to one’s satisfaction with their own body size or specific body parts. The socio-cultural environment seems to be an important variable in the development of distortions and subjective body image disorders.(6)

Identifying and measuring the magnitude of body image self-perception distortions would be relevant for the clinical evaluation of those individuals at risk of developing obesity. In that regard, the contour drawings scale is a highly effective instrument for assessing people’s level of dissatisfaction with their body weight and body size while evaluating the perceptual component of body image.(7) It is a helpful instrument for exploring ideal body image and objective image, particularly among overweight and obese individuals or those struggling to maintain their weight and control their eating behaviors.

The body mass index (BMI), or Quetelet index [body weight (kg)/height ^2^ (m)], is the most common measure used in population-based studies for primarily categorizing nutritional status.(8) Likewise, research assessing body image self-perception has used BMI as an indicator of nutritional status associated with determinants of body weight-related behaviors.(9)

For preventing and reducing excess weight, the efficiency and efficacy of strategies to be designed and applied in the clinical practice and for disseminating information to the general population rely on one’s realistic perception and self-awareness of their own body based on a real body size. This can be a valuable instrument in outpatient settings as an additional tool for clinically evaluating those seeking professional help due to body weight concerns.

However, most Brazilian studies on body image have used contour drawing scales developed and validated elsewhere for assessing individuals with different biotypes from Brazilian ones.(10)

The failure to accurately recognize their own overweight status prior to becoming obese may prevent them from changing behaviors that might contribute to additional weight gain. Therefore, it is important to understand the magnitude of weight status distortions within persons with BMI scores within the overweight range.(11)

This study was conducted with the objectives of knowing body mass index of female medical students, to find out their pattern of body image perception based on contour drawing scale and visual anologue scale, image satisfaction and any attempts was done by them to change their weight.

## Materials and Methods

The study was carried out in MBBS ladies hostel, Father Muller Medical College, Mangalore. All the 147 female medical students residing in the hostel were included in the study. Study was done during September and October 2007.

All the students were interviewed based on a questionnaire along with the measurement of height and weight. The questionnaire included questions regarding their perception of image, image satisfaction, diet, physical activity and any attempts done to change their weight. Image perception was measured using contour drawing scale and visual analogue scale.(12) There were nine figures of females used in the study. First three were lean, next three were normal, and the last three were overweight. Students were asked which body contour they matched. BMI was classified using WHO classification.(2) Informed consent was taken at the beginning of the study. Their responses were collected, tabulated, analyzed and interpreted.

Analysis was done by Chi-square tests.

## Results

Table 1 shows the distribution of study group according to age, BMI, body image perception, image satisfaction, attempts done to change weight and skipping meals as dieting. Out of 147 subjects studied 17 (11.6%) were 18 years of age, 23 (15.6%) were 19 years of age, 20 (13.6%) were 20 years old. About 35 (23.8%) were of 21 years, another 29 (19.5%) were of 22 years, while 23 (15.6%) belong to above 23 years of age.

**Table 1:** Distribution of study group according to age, BMI, body image perception, image satisfaction, attempts done to change weight

Age in years	Frequency(*n*=147)	%
18	17	11.6
19	23	15.6
20	20	13.6
21	35	23.8
22	29	19.7
>23	23	15.6
BMI- Kg/ m^2^		
Underweight(<18.50)	25	17.0
Normal(18.50-24.99)	111	75.5
Overweight/pre-obese(>25.00-29.99)	11	7.5
Obese(>30.00)	0	0.0
Body image perception		
Lean	35	23.8
Normal	95	64.6
Overweight	17	11.6
Image satisfaction		
Satisfied	98	66.7
Unsatisfied	49	33.3
Attempts done		
No attempt	98	66.7
Reduce weight	30	20.4
Skipping meals as dieting		
Skipping	42	28.6
Not skipping	105	71.4

Among the total 147 subjects, about 25 were underweight (17%), while 111 (75.5%) belong to normal range of BMI and 11 were overweight (7.5%). With reference to body image perception, about 35 (23.8%) of the subjects felt they were lean, a majority 95 (64.6%) felt they were normal and 17 (11.6%) felt they were overweight. Regarding image satisfaction, 98 (66.7%) were satisfied with their image while 49 (33.3%) were unsatisfied. Out of the 49 subjects who were unsatisfied, (20.4%) wanted to reduce weight. Skipping meals was practiced by 42 (28.6%) of the subjects.

Table 2 shows distribution of body image perception by age. Most of the females 95 (64.6%) felt they were normal. Only 17 (11.6%) of them felt they were overweight. This difference was not statistically significant.

**Table 2:** Distribution of image perception according to age of female students

Age	Image perception			Total No. (%)	
	Lean No. (%)	Normal No. (%)	Overweight No. (%)
18	6(35.3)	10(58.8)	1(5.9)	17(100.0)	χ^2^=13.113,
19	6(26.3)	13(56.5)	4(17.4)	23(100.0)	df=10,
20	8(40.0)	11(55.0)	1(5.4)	20(100.0)	*P*=0.217
21	4(11.4)	26(74.3)	5(14.3)	35(100.0)
22	7(24.1)	21(72.4)	1(3.4)	29(100.0)
>23	4(17.4)	14(60.9)	5(21.7)	23(100.0)

Total	35(23.8)	95(64.6)	17(11.6)	147(100.0)

Table 3 shows image perception of female students according to BMI class. Out of the 25 underweight female subjects, about 17 (68%) felt they were lean and 8 (32%) felt they had a normal image perception. Out of the 111 females who had a normal BMI, about 18 (16.2%) felt they were lean, 86 (77.5%) perceived their image as normal and 7 (6.3%) felt they were overweight. Similarly, out of the 11 who were overweight 1 (9.1%) felt normal, and 10 (90.9%) felt they were overweight. This association was found to be statistically significant.

**Table 3:** Distribution of the image perception according to BMI class of women

BMI class (Kg/ m^2^)	Image perception			
	Lean No. (%)	Normal No. (%)	Overweight No. (%)	Total No. (%)
Underweight	17(68.0)	8(32.0)	0(0.0)	25(17.0)
Normal	18(16.2)	86(77.5)	7(6.3)	111(75.5)
Overweight	0(0.0)	1(9.1)	10(90.9)	11(7.5)

Total(%)	35(23.8)	95(64.6)	17(11.6)	147(100.0)

χ^2^=103.442, df=4, *P*=0.0001

Table 4 shows distribution of image satisfaction according to BMI class of the female students. About 12 (48%) of the underweight females were satisfied while 13 (52%) were not satisfied with their body image perception. Among the 111 females with the normal BMI, around 86 (77.5%) were satisfied while 25 (22.5%) were not satisfied with the image. The proportion of females not satisfied with the image was higher with the overweight females. This difference was found to be statistically significant.

**Table 4:** Distribution of image satisfaction according to BMI class of the women

BMI class	Image satisfaction		
	Satisfied No. (%)	Unsatisfied No. (%)	Total No.(%)
Underweight	12(48.0)	13(52.0)	25(17.0)
Normal	86(77.5)	25(22.5)	111(75.5)
Overweight	0(0.0)	11(100.0)	11(7.55)

Total(%)	98(66.7)	49(33.3)	147(100.0)

χ^2^=31.758, df= 2, *P*=0.001

Table 5 shows the distribution of image satisfaction according to image perception of women. Out of the 35 subjects who felt they were lean by body image perception, 20 (57.1%) were satisfied while 15 (42.9%) were not satisfied with their image perception. Of the total 95 female subjects who perceived a normal image of themselves, a majority 76 (80%) were satisfied while 19 (20%) were not satisfied with their image perception. About 2 (11.8%) of the study subjects felt they were overweight were satisfied while 15 (88.2%) were not satisfied with their body image perception. This difference was found to be statistically significant (Chi square=32.807, *P*=0.001).

**Table 5:** Distribution of image satisfaction according to image perception of women

BMI class	Image satisfaction		
	Satisfied No. (%)	Unsatisfied No. (%)	Total No.(%)
Lean	20(57.1)	15(42.9)	35(23.8)
Normal	76(80.0)	19(20.0)	95(64.6)
Overweight	2(11.8)	15(88.2)	17(11.6)

Total(%)	98(66.7)	49(33.3)	147(100.0)

χ^2^= 32.807, df=2, *P*=0.001

Table 6 shows distribution of attempts to change weight according to image perception if image perceived was unsatisfied. All the females who felt that they were lean wanted to increase their weight; on the other hand, 15 (100%) females who felt they were overweight wanted to reduce their weight. Among the normal category, 15 (78.9%) wanted to reduce the weight while 4 (21.1%) wanted to gain weight. This difference was found to be statistically significant.

**Table 6:** Distribution of attempts to change weight according to image perception (if image unsatisfied

Image perception	Attempts done		
	Reduce weight No. (%)	Increase weight No. (%)	Total No.(%)
Lean	0(0.0)	15(100.0)	15(30.6)
Normal	15(78.9)	4(21.1)	19(38.8)
Overweight	15(100.0)	0(0.0)	15(30.6)

Total(%)	30(61.2)	19(38.8)	49(100.0)

χ^2^=35.7,df=2,*P*=0.001

Table 7 shows distribution of skipping meals according to image perception. Among the 35 lean subjects, 9 (25.7%) of the females had skipped meals while 26 (74.3%) did not skip meals. Among the 95 females who felt they had normal image, about 23 (24.2%) had skipped meals while a majority 72 (75.8%) did not skip meals. Among the 17 females in overweight category, around 10 (58.9%) had skipped meals, while 7 (41.1%) did not skip meals. This difference was found to be statistically significant.

**Table 7:** Distribution of skipping meals according to image perception

Image perception	Skipping meals		
	Skipped No. (%)	Not skipped No. (%)	Total No.(%)
Lean	9(25.7)	26(74.3)	35(23.8)
Normal	23(24.2)	72(75.8)	95(64.6)
Overweight	10(58.9)	7(41.1)	17(11.6)

Total(%)	42(28.6)	105(71.4)	147(100.0)

χ^2^= 8.649, df=2, *P*=0.013

## Discussion

In the present study BMI was classified among 147 subjects, 25 were underweight (17%), 111 were normal (75.5%) and 11 were overweight (7.5%) [Table 1]. So, majority were within normal range of BMI and overweight were fewer in number than the underweight. A study was done on first year students at Medical University of Silesia(13) which involved 518 females and 323 males. The level of overweight was estimated by the use of BMI and the prevalence of overweight was found to be 9.6%. The prevalence of overweight among males was two times greater than the prevalence among females (15.2% *Vs* 5.8%).

In the present study visual drawing scale was used and based on the body image perception 17 (68%) of underweight females felt they were lean and eight (32%) of them felt normal. About 86 (77.5%) of the females within normal range of BMI perceived their image as normal. Similarly one of overweight felt normal and 10 (90.9%) of them felt overweight. This may imply that a majority may be conscious of the effects of obesity. A study conducted to assess the relationship between body mass index and self-perception of body image by Kakesita *et al*.(12) showed that both men and women had a distorted self-perception of body image, underestimating or overestimating it. The study results suggest dissatisfaction of subjects with their body image as they desire to have leaner bodies. In their study, a Contour Drawing Rating Scale and Visual Analogue Scale were used to evaluate body image perception. In another study on body image perception, Sinead McElhone *et al* reported that 39% of the respondents in the European Union and about 29% Finnish were content with their weight. The highest percentage of subjects who were content with their body weight was among the females who were underweight (58%) and males who were normal weight (66%).(14) But in our study we found that majority perceived their image correctly.

In the present study all the underweight females wanted to increase weight. About nine of the overweight and 12 of the females with normal image perception wanted to reduce their weight. In a study by Hassapidou M *et al* on food choice criteria according to body image satisfaction among adolescents showed that around 39% (45% females and 26.6% males) wanted to lose weight, while 14% wished to gain weight after perception of their body image. The remaining 50% wanted to maintain their same body weight.(15)

In the present study skipping meals was practiced by students to reduce weight. Among 147 students who skipped meals were 42 (28.6%). Proportion of students who skipped meals was more among those who were overweight.

In a study to determine eating patterns and demographic and dietary factors associated with adolescent’s attempts to change weight,(16) data from students participating in the Massachusetts Youth Risk Behavior Survey (YRBS) were analyzed. A total of 61.5% of the females and 21.5% of the males reported trying to lose weight; 6.8% of the females and 36.3% of the males were trying to gain weight. There was a strong correlation (*r*=0.62, *P*<0.0001) between attempting to gain weight and self-perception of underweight for both genders. Females reported having changed their intake of several foods if attempting to change weight.

In a study conducted by Linde *et al*.(17) correlates of body mass index (BMI) in overweight and obese members of a managed care organization seeking treatment for obesity, they assessed intake of specific foods, dietary fat or fiber and behaviors attempted to control weight. Subscribing to exercise magazines decreased fat intake and increased fruit/ vegetable/fiber intake over the course of the study were associated with reductions in BMI. In our study as the students had less choice of food items in the hostel mess, they preferred skipping of meals as a method of choice to lose weight.

In the present study, around 75.5% of the study group were having normal BMI. Most of them perceived their image correctly regarding their weight. Most of the underweight and all overweight females were not satisfied with their body image. Underweight females preferred to gain weight. On the other hand, overweight females preferred to lose weight. Overweight students preferred skipping of meals as a means of losing weight.

## Conclusion

It was a study of assessment of body mass index, body image perception and attempts to change weight among the female medical students. Body image was perceived correctly by the female medical students and in-turn they had attempted to modify their body weight status toward normal.
